# Prediction of cell cycle distribution after drug exposure by high content imaging analysis using low‐toxic DNA staining dye

**DOI:** 10.1002/prp2.1203

**Published:** 2024-04-29

**Authors:** Kazuma Takeuchi, Yumiko Nishimura, Takayoshi Matsubara, Sho Isoyama, Asuka Suzuki, Masaaki Matsuura, Shingo Dan

**Affiliations:** ^1^ Division of Molecular Pharmacology, Cancer Chemotherapy Center Japanese Foundation for Cancer Research Tokyo Japan; ^2^ Graduate School of Public Health Teikyo University Tokyo Japan; ^3^ Division of Cancer Genomics, Cancer Institute Japanese Foundation for Cancer Research Tokyo Japan

**Keywords:** cell cycle, cell line, high‐content imaging analysis, machine learning

## Abstract

Interference in cell cycle progression has been noted as one of the important properties of anticancer drugs. In this study, we developed the cell cycle prediction model using high‐content imaging data of recipient cells after drug exposure and DNA‐staining with a low‐toxic DNA dye, SiR‐DNA. For this purpose, we exploited HeLa and MCF7 cells introduced with a fluorescent ubiquitination‐based cell cycle indicator (Fucci). Fucci‐expressing cancer cells were subjected to high‐content imaging analysis using OperettaCLS after 36‐h exposure to anticancer drugs; the nuclei were segmented, and the morphological and intensity properties of each nucleus characterized by SiR‐DNA staining were calculated using imaging analysis software, Harmony. For the use of training, we classified cells into each phase of the cell cycle using the Fucci system. Training data (*n* = 7500) and validation data (*n* = 2500) were randomly sampled and the binary classification prediction models for G1, early S, and S/G2/M phases of the cell cycle were developed using four supervised machine learning algorithms. We selected random forest as the model with the best performance through 10‐fold cross‐validation; the accuracy rate was approximately 75%–87%. Regarding feature importance, variables expected to be biologically related to the cell cycle, for example, signal intensity and nuclear size, were highly ranked, suggesting the validity of the model. These results showed that the cell cycle can be predicted in cancer cells by simply exploiting the current prediction model using fluorescent images of DNA‐staining dye, and the model could be applied for the use of future ex vivo drug sensitivity diagnosis.

## Graphical abstract

The top 10 variables in feature importance of the G1 prediction model and S/G2/M prediction model in HeLa cells (A, B), and those in MCF7 cells (C, D).

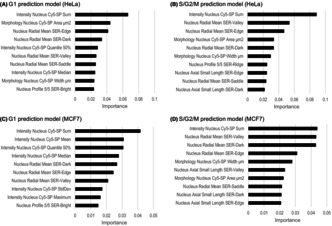

**Abbreviations**
Fucci: fluorescent ubiquitination‐based cell cycle indicator
G1: Gap 1 phase
G2: Gap 2 phase
HCA: high‐content imaging analysis
M: mitotic phase
mAG: monomeric Azami‐Green
mKO2: monomeric Kusabira‐Orange2
ROI: region of interest
S: synthesis phase

## 1 INTRODUCTION

Bioactive compounds exert some effect on living cells, causing phenotypic changes. Among these, those that are found to affect the proliferation and viability of cancer cells may become anticancer agents. The cell cycle has long been a focus of attention and remains an important target, as many compounds are known to show anticancer activity by affecting the cell cycle distribution of cancer cell populations.^1^ Flow cytometry is often used to observe cell cycle progression, but it requires fixing the cells and nuclear staining with a DNA‐staining dye such as propidium iodide. In contrast, the development of a fluorescent ubiquitination‐based cell cycle indicator (Fucci) has made it possible to observe the cell cycle in real time using a fluorescent microscope. Fucci is a fluorescent probe that visualizes cell cycle progression by fusing a red fluorescent protein called monomeric Kusabira‐Orange2 (mKO2) or mCherry to Cdt1 (Cdc10 dependent transcript), which is abundantly expressed in Gap 1 phase (G1) and degraded in S phase, and a green fluorescent protein called monomeric Azami‐Green (mAG) to Geminin, which is abundantly expressed in S phase and degraded in late mitotic phase (M phase) to early G1 phase.^2^ However, it takes some effort to introduce Fucci constructs into a cell line with lentiviral vectors and make them available for experimental use, and sometimes it is particularly difficult to perform lentiviral introduction to primary tumor cells.

The development of a high‐content imaging system in combination with the low‐toxic DNA‐staining dye SiR‐DNA^3^ enables time‐lapse imaging of cultured cells to obtain real‐time high‐content imaging data such as cell nucleus area, fluorescence intensity, and texture analysis.^4^ In this study, we aimed to develop a prediction model of the cell cycle using high‐content imaging data from SiR‐DNA staining.

## 2 MATERIALS AND METHODS

### 2.1 Experiments

Fucci‐expressing HeLa cells (namely HeLa/Fucci(RCB2812))^2^ and Fucci‐expressing MCF7 cells (MCF7/Fucci(SA)2.5)^5^ were seeded in 384‐well plates at a density of 1000 cells/well and preincubated in the presence of 0.25 μM SiR‐DNA (Cytoskeleton, Inc., Denver, CO) for 2 h at 37°C in a humidified atmosphere of 5% CO2. We then added a dilution series (five concentrations ranging from 10^−10^ to 10^−4^ M) of the test compounds: 5‐FU, SN‐38 (Sigma Aldrich Japan, Tokyo, Japan); selumetinib (Selleck Biotech, Tokyo, Japan); staurosporine (Fujifilm Wako Pure Chemical Corporation, Osaka, Japan); and ZSTK474 (Zenyaku Kogyo, Co., Ltd., Tokyo, Japan). Fluorescent images of SiR‐DNA‐stained cells, as well as bright‐field images, were acquired 36 h after drug exposure using the Operetta CLS high‐content imaging system with a 10x objective lens (Revvity, Inc., Waltham, MA). The Wavelengths of excitation LEDs/emission filters used are: 550/570–650 nm for mKO2 (or mCherry)‐hCdt1; 475/500–550 nm for mAG‐hGeminin; and 630/655–760 nm for SiR‐DNA, respectively.

### 2.2 Image analyses using Harmony software

The captured images were analyzed using Harmony software (Revvity). First, we applied a sliding parabola (SP) filter to reduce background noise, and nuclei were segmented using the *FindNuclei* building block. Basic morphology (i.e., area, roundness, width, and length) and intensity properties (i.e., mean, standard deviation, median, max, min, sum, coefficient of variance, quantile‐50%, and contrast) of each ROI (region of interest) of the segmented nucleus were calculated using the *Calculate Intensity Properties* (Standard) building block. To capture morphological properties, we utilized *the Calculate Morphology Properties* (Standard and STAR) and *Calculate Texture Properties* (SER and Haralick) building blocks. The SER (Spots, Edges, and Ridges) method uses eight different filters (bright, ridge, dark, saddle, edge, spot, hole, and valley) that highlight different patterns of staining intensity. The STAR method was used to calculate morphological properties (i.e., symmetry, threshold compactness, axial, radial, and profile) for each ROI. When running the STAR method, either of eight SER filters were applied. Through these analyses, a total of 240 numerical parameters were generated. Detailed phenotypic profiles using texture and advanced morphology analyses are described elsewhere.^6^

### 2.3 Data preprocessing for supervised machine learning

In this study, we used complete data without missing measurements. We determined the thresholds from the distribution of mean fluorescence intensity within the segmented ROI with respect to the two Fucci colors to be used as explanatory variables for classifying nuclei into four groups, G1, early S, S/G2/M, and double negative (DS). For HeLa/Fucci cells, we defined G1 phase: mKO2 ≧700/mAG < 2500; early S phase: mKO2 ≧700/mAG ≧2500; S/G2/M phase: mKO2 < 700/mAG ≧2500; DS: mKO2 < 700/mAG < 2500, respectively. For MCF7/Fucci cells, we defined G1 phase: mCherry ≧150/mAG < 300; early S phase: mCherry ≧150/mAG ≧300; S/G2/M phase: mCherry <150/mAG ≧300; DN: mCherry <150/mAG <300, respectively. In addition, apoptotic nuclei with CV values greater than 70% were excluded to avoid the unfavorable effects of substantial contamination of dead cells in MCF7 experiments. Then nuclei for training (*n* = 7500) and validation (*n* = 2500) were extracted by random sampling. In order to unify the scale among the explanatory variables, normalization was performed so that the data fit between 0 and 1 based on the minimum and maximum values.

### 2.4 Construction of cell cycle prediction model

We constructed a binary classification prediction model with three patterns defined from Fucci's information: “G1” or “else,” “early S” or “else,” and “S/G2/M” or “else.” The following four models were used in this study: logistic regression as a representative model of classification models in statistical methods; support vector machine (SVM), which was used mainly before the advent of deep learning and still has relatively high classification performance; random forest, a model that creates a large number of decision trees in parallel; and neural network (3 hidden layers of a sequential model), a model that is considered to be the basic model of deep learning. The model development methods are described in the Data S1. We developed these models and compared their performance by performing a 10‐fold cross validation. The validation data were then used to evaluate the model performance of the selected models. Accuracy, sensitivity, specificity, and Area Under the Curve were calculated as evaluation indices. Additionally, we calculated the importance of the explanatory variables for the selected models. Histograms were drawn for the variables with the highest importance for each group defined by Fucci, and together with the importance of the explanatory variables, the validity of the predictive models was confirmed.

## 3 RESULTS

### 3.1 Development of cell cycle prediction models using high‐content imaging data with SiR‐DNA staining

To develop cell cycle prediction models using high‐content imaging data after cell staining with SiR‐DNA, we used two Fucci‐expressing cancer cell lines, HeLa/Fucci and MCF7/Fucci. We first obtained fluorescent images of cells under the indicated experimental conditions (Figure S1) and prepared a dataset for supervised machine learning. In brief, nuclei were segmented, and 240 numerical parameters characterizing the morphological properties of each nucleus were generated by imaging analysis using SiR‐DNA‐stained images. At the same time, the segmented nuclei were classified into each phase of the cell cycle using the Fucci system. We then developed binary classification prediction models for each cell cycle phase (“G1” or “else”; “early S” or “else”; “S/G2/M” or “else”) using the 240 parameters from the training data (*n* = 7500) by exploiting four machine learning algorithms as described in materials and methods. A comparison of the accuracy rates of the HeLa prediction models in a 10‐fold cross‐validation revealed that the random forest model exhibited the highest accuracy of all models used for G1‐, early S‐, and S/G2/M prediction (Table 1, upper panel). Interestingly, similar results were observed in another dataset obtained from experiments using MCF7/Fucci cells.

**TABLE 1:** Accuracy of five prediction models by 10‐fold cross‐validation (upper panel) and the evaluation of random forest model using validation data (lower panel).

Cell line	HeLa			MCF7		
Prediction model	G1	Early S	S/G2/M	G1	Early S	S/G2/M
Cross‐validation^a^						
Logistic regression (all variables)	0.751	0.539	0.839	0.731	0.593	0.751
Logistic regression (backward stepwise)	0.761	0.608	0.838	0.733	0.612	0.763
Support‐vector machine	0.753	0.829	0.856	0.715	0.627	0.770
Deep neural network	0.743	0.707	0.840	0.710	0.623	0.783
Random forest	0.777	0.833	0.867	0.755	0.746	0.829
Model evaluation^b^ (random forest)						
Accuracy	0.772	0.834	0.836	0.746	0.735	0.832
Sensitivity	0.787	0.064	0.579	0.828	0.424	0.695
Specificity	0.761	0.997	0.965	0.622	0.809	0.859
Area under the curve (AUC)	0.855	0.671	0.913	0.805	0.690	0.858

Abbreviations: G1, Gap 1 phase; G2, Gap 2 phase; M, mitotic phase; S, synthesis phase.

^a^ Training data were used for 10‐fold cross‐validation. Evaluation indices are the percentages of accuracy.

^b^ The accuracy, sensitivity, specificity, and AUC of the random forest model were evaluated using validation data.

Using the validation dataset (*n* = 2500), we evaluated the prediction performance of the selected random forest models and found that they exhibited preferable scores, except for the models predicting early S with extremely low sensitivity (Table 1, lower panel). Therefore, we decided to use the G1 and S/G2/M prediction models in the subsequent analysis.

### 3.2 Cell cycle prediction in cell population after exposure to antitumor agents

To evaluate the cell cycle prediction performance after drug exposure, the breakdown of the nuclei counts predicted as “G1” or “else,” “S/G2/M” or “else,” along with the count in each phase of the cell cycle classified by the Fucci system, is shown in Figure S2. In this study, we selected five anticancer compounds with various modes of action: an antimetabolite 5‐FU; a MEK1 inhibitor selumetinib; a DNA‐topoisomerase I inhibitor SN‐38; a multikinase inhibitor/potent apoptosis inducer staurosporine; and a PI3K inhibitor ZSTK474. Since nuclei in the validation dataset (*n* = 2500) were randomly sampled, the numbers of nuclei under the respective experimental conditions roughly reflect the number of cells and the distribution of the cell cycle. Overall, the accuracy of the prediction in both HeLa and MCF7 cells after drug exposure is comparable to that in control cells, except for the cell population after exposure to high doses of cytotoxic drugs; for example, staurosporine at 100 nM or higher. Notably, HeLa cells accumulated in the S/G2/M phase and those in the G1 phase were obviously reduced in samples exposed to 5‐FU (10 μM or higher) and SN‐38 (0.1 μM or higher), as visualized by the Fucci system, and similar distribution changes are predicted, as shown in the size of pie charts (Figures S1A and S2A). Moreover, such distribution changes were also captured in the S/G2/M prediction model (Figure S2B). In contrast, MCF7 cells did not accumulate in the S/G2/M phase classified by the Fucci system, nor did they in the prediction. Instead, MCF7 cells accumulated in the G1 phase after exposure to ZSTK474 (1 μM or more), as they did in the prediction, whereas such accumulation was not observed, or predicted in HeLa cells (Figure 2A,C).

### 3.3 Importance of explanatory variables in the selected random forest prediction model

**FIGURE 1:**
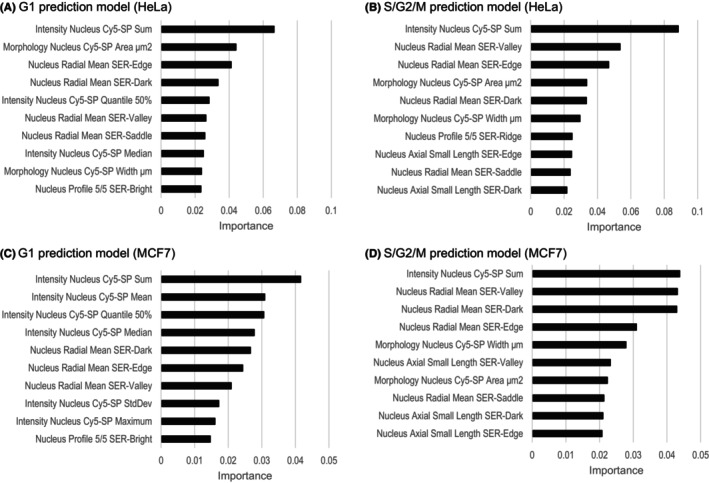
The top 10 variables in feature importance of the random forest model selected in cross‐validation. Feature importance of the G1 prediction model (A), S/G2/M prediction model (B) in HeLa cells, G1 prediction model (C), and S/G2/M prediction model (D) in MCF7 cells. G1, Gap 1 phase; G2, Gap 2 phase; M, mitotic phase.

**FIGURE 2:**
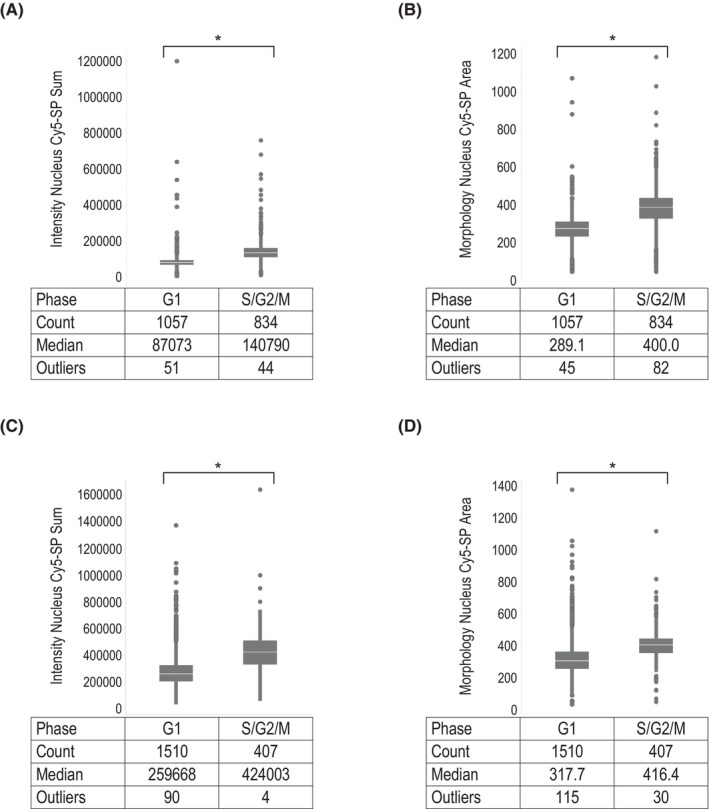
Box plot of total fluorescence intensity and nuclear area at the top of feature importance using validation data. Box plot analyses of fluorescence intensities (A, C) and areas (B, D) of the nuclei classified in G1‐ and S/G2/M populations by Fucci system in HeLa (A, B) and MCF7 (C, D) dataset. The *p*‐value was calculated by the Wilcoxon rank sum test. *Indicates *p* < .001. G1, Gap 1 phase; G2, Gap 2 phase; M, mitotic phase.

We next examined the importance of explanatory variables in the selected random forest prediction model. As shown in Figure 1, the variables expected to reflect the DNA content of the cells, for example, signal intensity and area size of the nucleus, were highly ranked in both the HeLa and MCF7 datasets. Actually, these parameters tended to have larger values in S/G2/M‐phase nuclei than in G1‐phase nuclei (Figure 2). In addition, some variables obtained with the *Calculate Morphology Properties* (STAR) building block using SER texture‐filtered images, that is, “Nucleus Radial Mean SER‐Edge,” “‐Valley,” and “‐Dark,” are also highly ranked in common, suggesting their usefulness in cell cycle prediction.

## 4 DISCUSSION

In this study, we demonstrated a cell cycle prediction model using a random forest algorithm by exploiting high‐content imaging parameters of SiR‐DNA‐stained tumor cells. Using this model, changes in cell cycle distribution after drug exposure in HeLa and MCF7 cells can be roughly estimated. We are now trying to develop a tumor type agnostic cell cycle prediction model. In order to achieve this, it will be necessary to examine various cell types other than HeLa and MCF7 cells to identify common explanatory variables for use in cell cycle prediction. Actually, major explanatory variables selected in the current prediction models with HeLa‐ and MCF7 datasets commonly included variables that are clearly biologically related to the cell cycle, such as the fluorescence intensity of nuclei. The identification of such variables will enable us to develop a versatile prediction model. In addition, some textural features such as “Nucleus Radial Mean SER‐Edge,” “‐Valley,” and “‐Dark” were also highly ranked in both datasets, suggesting their usefulness in cell cycle prediction. Texture analysis is expected to play an important role in extracting meaningful morphological features, and the radial mean is the mean object radius based on the intensity value weighted by the distance from the mass center; however, the causal relationships between these features and cell cycle progression, as well as the biological meaning of these textural features, remain to be elucidated.

In summary, the current prediction model allows us to estimate the effect of anticancer drugs on cell cycle in HeLa and MCF7 cell lines, and identification of common features related to cell cycle progression suggested the possibility of a versatile prediction model by increasing recipient tumor cell lines used for training and fine‐tuning the prediction model. By realizing these, it will be possible to apply them to patient‐derived cells to diagnose their anticancer drug sensitivity ex vivo.

## AUTHOR CONTRIBUTIONS

Participated in research design: Kazuma Takeuchi, Yumiko Nishimura, Masaaki Matsuura, and Shingo Dan. Conducted experiments: Kazuma Takeuchi, Yumiko Nishimura, Sho Isoyama, and Takayoshi Matsubara. Performed data analysis: Kazuma Takeuchi, Yumiko Nishimura, Takayoshi Matsubara, Sho Isoyama, Asuka Suzuki, and Masaaki Matsuura. Wrote or contributed to the writing of the manuscript: Kazuma Takeuchi, Masaaki Matsuura, and Shingo Dan.

## FUNDING INFORMATION

This work was supported in part by Grants‐in‐Aid for Scientific Research (B) from Japan Society for the Promotion of Science (JSPS KAKENHI Grant Number 19H03525), the Molecular Profiling Committee, the Grant‐in‐Aid for Transformative Research Areas “Advanced Animal Model Support (AdAMS)” from the Ministry of Education, Culture, Sports, Science and Technology, Japan (JSPS KAKENHI Grant Number JP 22H04922), and a grant from the Nippon Foundation.

## CONFLICT OF INTEREST STATEMENT

The authors declare no conflict of interest.

## ETHICS STATEMENT

This study using established human cancer cell lines does not require ethics committee approval procedures; studies using the Fucci‐introduced cell lines were conducted in an environment compliant with biosafety levels under the approval of the Genetic Recombination Safety Committee of the Japanese Foundation for Cancer Research.

## Supporting information


Figure S1.
Figure S2.

Data S1:

## Data Availability

The raw image data that support the findings of this study are available from the corresponding author upon reasonable request.
