# Supplementary material for: Prediction of cell cycle distribution after drug exposure by high content imaging analysis using low‐toxic DNA staining dye
Source: Pharmacol Res Perspect. 2024 Apr 29;12(3):e1203. doi: 10.1002/prp2.1203 (PMC11057241; doi:10.1002/prp2.1203)
Supplement: Supplementary file 2 — Data S1: [file PRP2-12-e1203-s002.docx]

Supplemental Methods

**Logistic regression**

We used two types of logistic regression models: one using all variables and the other using the stepwise Backward Elimination method. The backward elimination method evaluates the p-values of each explanatory variable after constructing an all-variable model, and repeats the process of eliminating and reevaluating the variables in order until there are no variables with a p-value below a significance level of 0.05. The API (Application Programming Interface) of the statsmodels package in Python (https://www.statsmodels.org/dev/api.html) was used for the logistic regression analysis.

**Support Vector Machine (SVM)**

Support vector machine is an algorithm that learn to draw a line so as to maximize the distance between this boundary line and the point closest to the line. For this purpose, SVM module of the Python scikit-learn library (https://scikit-learn.org/stable/ modules/generated/sklearn.svm.SVC.html) was used. Among the hyperparameters whose values must be specified, kernel (the type of kernel used in the algorithm), cost parameter C (a parameter that represents the severity of the regularization term and soft margin), RBF (Gaussian) kernel parameter γ (a parameter that adjusts the nonlinear decision boundary by the kernel trick) were determined by Bayesian optimization, using the Python Optuna library (https://optuna.readthedocs.io/en/stable/index.html).

**Random Forest**

Random forest is a method of creating multiple decision trees with different contents in parallel and deciding the final result by majority voting on the predicted results of those decision trees. In this study, we use the RandomForestClassifier of the ensemble module of the Python scikit-learn library (https://scikit-learn.org/stable/modules/generated/sklearn. ensemble.RandomForestClassifier.html) was used. Among the hyperparameters whose values must be specified, n_estimators (number of decision tree models), max_depth (maximum decision tree depth), min_samples_split (number of samples required for node splitting), min_samples_leaf (number of samples required for leaves after splitting), and max_features (number of variables and selection criteria) were determined by the optimization methods using Bayesian theory-based stochastic models with the Python Optuna library (https://optuna.readthedocs.io/en/stable/index.html).

**Neural network (hidden layer: 3)**

The neural network was constructed using the Sequential model (https://keras.io/ja/getting-started/sequential-model-guide/) from the Python Keras library. The input layers were the number of variables in each data; the number of hidden layers is 3; the number of neurons in the hidden layers was 128, and the activation function to the hidden layer was the ReLU function. The output layer was one layer, and the activation function from the hidden layer to the output layer was a sigmoid function. In addition, Adam was used as the optimization model, and binary cross-entropy was used as the loss function. A 10% dropout was randomly applied to neurons in each layer as a precautionary measure against overlearning. Youden index was used as the cutoff for binary discrimination in this model.
